# Duplication and diversification of insulin genes in ray-finned fish

**DOI:** 10.24272/j.issn.2095-8137.2018.052

**Published:** 2019-05-18

**Authors:** David M. Irwin

**Affiliations:** 1 Department of Laboratory Medicine and Pathobiology, University of Toronto, Toronto Ontario M5S 1A8, Canada; 2 Banting and Best Diabetes Centre, University of Toronto, Toronto Ontario M5S 1A8, Canada

**Keywords:** Insulin, Teleost fish, Gene duplication, Adaptive evolution, Gene loss

## Abstract

Insulin is a key hormone for the regulation of metabolism in vertebrates. Insulin is produced by pancreatic islet cells in response to elevated glucose levels and leads to the uptake of glucose by tissues such as liver and adipose tissue to store energy. Insulin also has additional functions in regulating development. Previous work has shown that the proglucagon gene, which encodes hormones counter regulating insulin, is duplicated in teleost fish, and that the peptide hormones encoded by these genes have diversified in function. I sought to determine whether similar processes have occurred to insulin genes in these species. Searches of fish genomes revealed an unexpected diversity of insulin genes. A triplication of the insulin gene occurred at the origin of teleost fish, however one of these three genes, *insc*, has been lost in most teleost fish lineages. The two other insulin genes, *insa* and *insb*, have been retained but show differing levels of selective constraint suggesting that they might have diversified in function. Intriguingly, a duplicate copy of the *insa* gene, which I named *insab*, is found in many fish. The coding sequence encoded by *insab* genes is under weak selective constraint, with its predicted protein sequences losing their potential to be processed into a two-peptide hormone. However, these sequences have retained perfectly conserved cystine residues, suggesting that they maintain insulin’s three-dimensional structure and therefore might modulate the processing and secretion of insulin produced by the other genes.

## INTRODUCTION

Fish have been important contributors to our understanding of human biology and disease (Lieschke & Currie, 2007; MacRae & Peterson, 2015), especially in endocrinology (Conlon, 2000a). In vertebrates, insulin is a hormone produced by the beta-cells of pancreatic islets in response to increased levels of blood glucose, which induces the uptake of glucose by tissues such as liver and adipose tissue for storage, and thus is a key regulator of glucose metabolism (Röder et al., 2016). Deficiencies in insulin production and/or signaling leads to diabetes in humans and other animals (Röder et al., 2016; Weiss, 2009). Many aspects of insulin function have been learned from extensive studies in diverse species of fish (Caruso & Sheridan, 2011; Conlon, 2000b; Polakof et al., 2012). Fish have also been explored in the development of potential treatments for diabetes. Xenotransplantation of fish islets, using both wild-type and humanized insulin, has been considered due to the relative ease of isolation of islets from pancreatic tissue in fish (e.g., tilapia, *Oreochromis niloticus*) compared to mammalian sources (Wright et al., 2014). Insulin, however, in addition to its role in the regulation of metabolism, also has other roles in vertebrate biology, including some in development (Chan & Steiner, 2000; Hernández-Sánchez et al., 2006).

Insulin is a polypeptide hormone composed of two peptides, A- and B-chains of about 20 and 30 amino acids, respectively, which are held together by disulfide bridges (Conlon, 2001; Steiner et al., 1985, 2009; Weiss, 2009). Insulin sequences have been characterized, mostly at the protein level, in a large number of vertebrate and fish species (Caruso & Sheridan, 2011; Conlon, 2000b, 2001). Typically, vertebrate species have a single copy of the insulin gene in their genomes (Nishi & Nanjo, 2011; Steiner et al., 1985), however, multiple insulin genes have been characterized in some species, such as rodents (Long et al., 2013; Soares et al., 1985; Wentworth et al., 1986) and some fish (Caruso et al., 2008; Caruso & Sheridan, 2011, 2014; Irwin, 2004). Within fish, it appears that only one of these insulin genes, which produces the hormone that regulates blood glucose levels, is expressed in the pancreas (Caruso & Sheridan, 2011; Irwin, 2004), with the exception of the multiple insulin genes produced by the very recent genome duplication in salmonid fish (Caruso et al., 2008; Caruso & Sheridan, 2014). While the origin of the two insulin genes found in some fish (e.g., zebrafish) was shown to be early in teleost fish evolution, whether this *ins2* gene has been retained in the genomes of diverse fish is unclear (Caruso & Sheridan, 2011; Irwin, 2004). The fish *ins2* gene has been poorly characterized. The initial identification of this gene in zebrafish (*Danio rerio*) and takifugu (*Takifugu rubripes*) only showed expression in the embryo of zebrafish (Irwin, 2004). A subsequence study of the expression of the two insulin genes in zebrafish suggested that *ins2* potentially has a function as a growth and neurotrophic factor during development (Papasani et al., 2006). More recently, studies of the tilapia (*O. niloticus*) *ins2* gene showed that it had negligible expression in the pancreas, thus likely is not a major contributor to the regulation of glucose metabolism and would not need to be silenced to allow the xenotransplantation of tilapia islets as a treatment for diabetes in humans (Hrytsenko et al., 2016).

Changes in the function of insulin can be paralleled by changes in the function of glucagon, the hormone that counters the action of insulin (Seino et al., 1988). Recently it has been shown that duplicated proglucagon genes are widespread in teleost fish, and that the functions of the proglucagon-derived peptides encoded by these genes have diversified (Irwin & Mojsov, 2018). Here I have taken advantage of the large number of fish genomes that have been characterized in the past few years (Ravi & Venkatesh, 2018) to determine the distribution of insulin genes in the genomes of ray-finned fish. Analysis of these sequences allows us to begin to understand the origin of these genes, including the measurement of the selective forces acting upon them, which might provide evidence for differences in function. Surprisingly, I found a greater diversity in the numbers of insulin genes than expected, with the identification of a third insulin paralog, as well as lineage-specific duplicates of some of these paralogs. My analyses suggest that all three fish insulin paralogs encode functional protein products.

## MATERIALS AND METHODS

### Data collection

**Figure 1:**
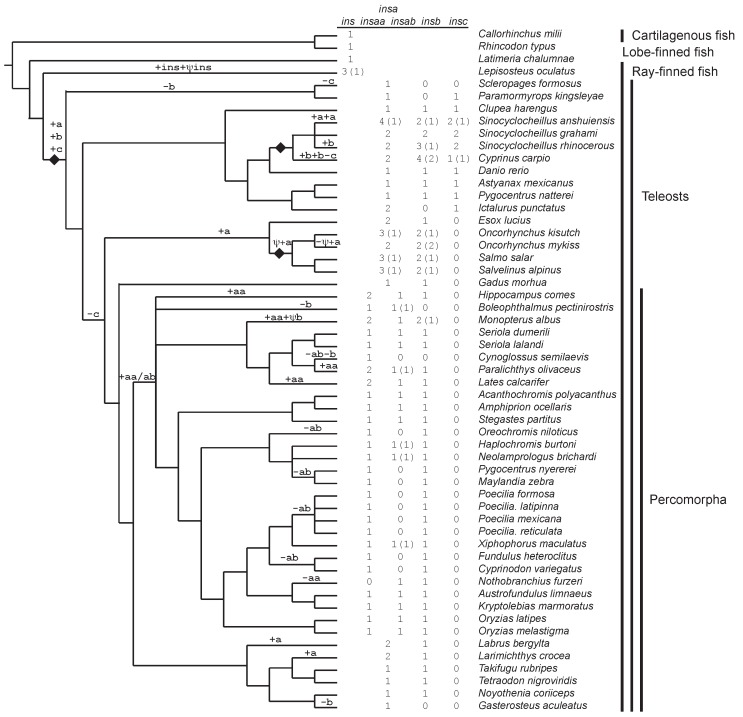
Diversity and evolution of fish insulin genes

Genome sequences databases maintained by the National Center for Biotechnology Information (NCBI: https://www.ncbi.nlm.nih.gov/genome/gdv/) and Ensembl (http://www.ensembl.org) were searched in March/April 2018, for sequences that were predicted to encode proteins similar to proinsulin. Initial searches used the *tBLASTn* algorithm (Gertz et al., 2006) using previously characterized zebrafish (*Danio rerio*) proinsulin protein sequences (Irwin, 2004; Milewski et al., 1988) as queries. Subsequent *tBLASTn*, *BLASTn*, and *BLASTp* searches used our putative proinsulin sequences as queries. Genome sequences of 55 fish (52 ray-finned (Superclass Actinopterygii), 2 cartilaginous (Superclass Chondrichthyes), and one lobe-finned (Class Sarcopterygii)) were examined, with 52 of these contained in the NCBI Genome Data Viewer, and three (Atlantic cod, *Gadus morhua*; three-spined stickleback, *Gasterosteus aculeatus*; and the spotted green pufferfish, *Tetraodon nigroviridis*) only in the Ensembl database (9 were in both NCBI and Ensembl). A list of species with genomes used here, and their phylogenetic relationships, is presented in Figure 1. All sequences with E-scores below 0.001 were examined. As an initial step to assess homology and orthology, reciprocal BLASTp searches of the zebrafish proteome were conducted to examine the similarity of the putative proinsulin sequences. Sequences that were not more obviously similar to insulin-like growth factor-I (*igf1*) or insulin-like growth factor-II (*igf2*) were used for the following analyses. Here I suggest a standardized set of names for fish *ins* genes, based on the analysis described below, with *insa*, *insb*, and *insc* representing the three *ins* paralogs that originated early in teleost fish evolution, and *insaa* and *insab* representing duplicates of the *insa* gene shared by multiple species. Arabic numerals are used for recent lineage-specific duplicates and do not indicate necessarily indicate orthology.

Summary of the evolution of insulin genes in ray finned fish. Phylogenetic relationships of species with genome sequences is based on Betancur-R et al. (2017), with modifications in an area that is not confidently resolved to minimize the number of gene duplications or deletions. Some phylogenetic relationships are not resolved. Branches are not proportional to evolutionary time. Classification of fish is shown to the right. Solid diamonds indicate genome duplications. The numbers of different types of insulin gene found in each genome is listed, with numbers in brackets indicating incomplete sequences. Gene additions or gene losses are indicated by plus (+) and minus (–) before an a, b, or c, representing the *insa*, *insb*, and *insc* paralogs, respectively. The *insa*, *insb*, and *insc* paralogs originated near the genome duplication in the ancestor of teleosts and are indicated as +a, +b, and +c to refer to the two gene duplications. The duplication to generate the *insaa* and *insab* genes is indicated by +aa/ab. +ins indicated the gene duplication on the *Lepisosteus oculatus* lineage. +ψins, +ψa, and +ψb indicates retroposition events that generated processed pseudogenes.

### Phylogenetic analysis

Prior to phylogenetic analysis, proinsulin (*ins*) coding sequences were aligned using the MAFFT algorithm (Katoh et al., 2001) as implemented at the Guidance2 server (http://guidance.tau.ac.il/ver2/; Penn et al., 2010). Phylogenetic relationships were established using both maximum likelihood and Bayesian methods. PhyML 3.0 (http://www.atgc-montpellier.fr/phyml/; Guindon et al., 2010) was used to generate maximum likelihood trees, with 500 bootstrap replications, where the Smart Model Selection (SMS) (Lefort et al., 2017) option was used to identify the best fitting evolutionary model. Bayesian trees were constructed using MrBayes 3.2.6 (Huelsenbeck et al., 2001; Ronquist et al., 2012), with 5 000 000 generations and four simultaneous Metropolis-coupled Monte Carlo Markov chains sampled every 100 generations under the same evolutionary model used for maximum likelihood. The first 25% of the trees were discarded as burn-in with the remaining samples used to generate consensus trees. Insulin coding sequences from cartilaginous (*Callorhinchus milli* (Elephant shark) and *Rhincodon typus* (Whale shark)) and lobe-finned fish (*Latimeria chalumnae* (Coelacanth)) were used as outgroups to root the trees for all insulin coding sequences. Subsequent analyses, using subsets of the sequence data, were conducted with similar approaches, and used sequences identified in our initial analyses as outgroups.

### Evolutionary analysis

Genes adjacent to insulin genes in fish genomes were identified by examining genomic contigs in the NCBI and Ensembl databases using methods that I have previously used (Irwin, 2002, 2012; Irwin & Mojsov, 2018). Briefly, the identity and orientation of genes neighboring insulin genes were identified

from the genomic databases. The orthology of the genes adjacent to insulin genes was assessed (or confirmed if annotated) using *BLASTp* searches of the zebrafish and human proteomes (Kuraku & Meyer, 2012).

The strength of selective pressure acting upon the insulin coding sequences can be measured by comparing the relative rates of nonsynonymous (*d*_N_) and synonymous substitutions (*d*_S_). Sequences under stronger selective pressure for protein function will have lower nonsynonymous to synonymous (*d*_N_/*d*_S_) ratios. *d*_N_/*d*_S_ rate ratios were obtained from analyses using RELAX (Wertheim et al., 2014) as implemented on the Datamonkey Adaptive Evolution server (http://datamonkey.org/; Weaver et al., 2018), which also tested for intensification or relaxation of the levels of selection on tested lineages. Evidence for positive selection on branches of the phylogenetic tree was tested using aBSREL (Smith et al., 2015) on the Datamonkey Adaptive Evolution server (Weaver et al., 2018).

### Prediction of protein processing sites and alignment of peptide sequences

Signal peptidase cleavage sites in the proinsulin protein sequences were predicted using SignalP 4.1 (http://www.cbs.dtu.dk/services/SignalP/; Petersen et al., 2011). I used two prediction programs to identify potential prohormone protease processing sites in the proinsulin sequences, NeuroPred (http://neuroproteomics.scs.illinois.edu/neuropred.htm; Southey et al., 2006) and ProP 1.0 (http://www.cbs.dtu.dk/services/ProP/; Duckert et al., 2004), with “general PC prediction” selected. To visual the conservation of the processing sites and insulin peptide sequences, the proinsulin protein sequences were aligned using Clustal Omega (https://www.ebi.ac.uk/Tools/msa/clustalo/; Sievers et al., 2011). Consensus proinsulin A- and B-chain peptide sequences were generated from the alignments using WebLogo 3 (http://weblogo.threeplusone.com/; Crooks et al., 2004).

## RESULTS

### Numbers of insulin genes in fish genomes

Searches of genome databases maintained by NCBI and Ensembl resulted in the identification of 168 insulin genes from the genomes of 55 species examined, with 141 of these predicted to be intact full-length coding sequences (Figure 1 and Supplementary Table S1). While only single copy insulin genes were found in the genomes of cartilaginous and lobe-finned fish, multiple genes were found in the genomes of most ray-finned fish. Within ray-finned fish, the number of identified insulin genes ranged from 1 (*Sclerophages fromosus*) to 8 (*Sinocyclocheilus anshuiensis*). As expected, fish species that experienced recent genome duplications, i.e., carp (*Cyprinus carpio*; Xu et al., 2014), members of the genus *Sinocyclocheilus* (Yang et al., 2016), and salmonids (*Oncorhynchus kisutch*, *O. mykiss*, *Salmo salar*, and *Salvelinus alpinus*; Lien et al., 2016) have the largest numbers of insulin genes. Phylogenetic analysis (see below) suggested the presence of three insulin paralogs in teleost fish: *insa*, *insb*, and *insc*. Of the 162 insulin genes identified in the 51 species of teleost fish (infraclass Teleostei), 92 were classified as *insa* (which includes *insaa* and *insab* genes), 57 as *insb*, and 13 as *insc*, with 11, 12, and 3 of the sequences found to be incomplete, respectively, for these three paralogs (Figure 1 and Supplementary Table S1). All 51 species of teleost fish possessed at least one *insa* gene, with 45 having *insb* genes, and 10 with *insc* genes (Figure 1 and Supplementary Table S1). The 6 species of teleost fish where I failed to identify *insb* genes are distributed across the accepted phylogeny of fish (Figure 1; Betancur-R et al., 2017; Near et al., 2012), suggesting they were lost independently several times (or are missing from these genome assemblies). In contrast, almost all of the species lacking *insc* genes share a common ancestor (Figure 1, clade Euteleostei), suggesting a single gene loss event explains the absence of *insc* genes in most teleost fish. The coding sequences of the insulin (*ins*) genes used in our analysis described below are presented in Supplementary Figure S1.

### Phylogeny of fish insulin genes

**Figure 2:**
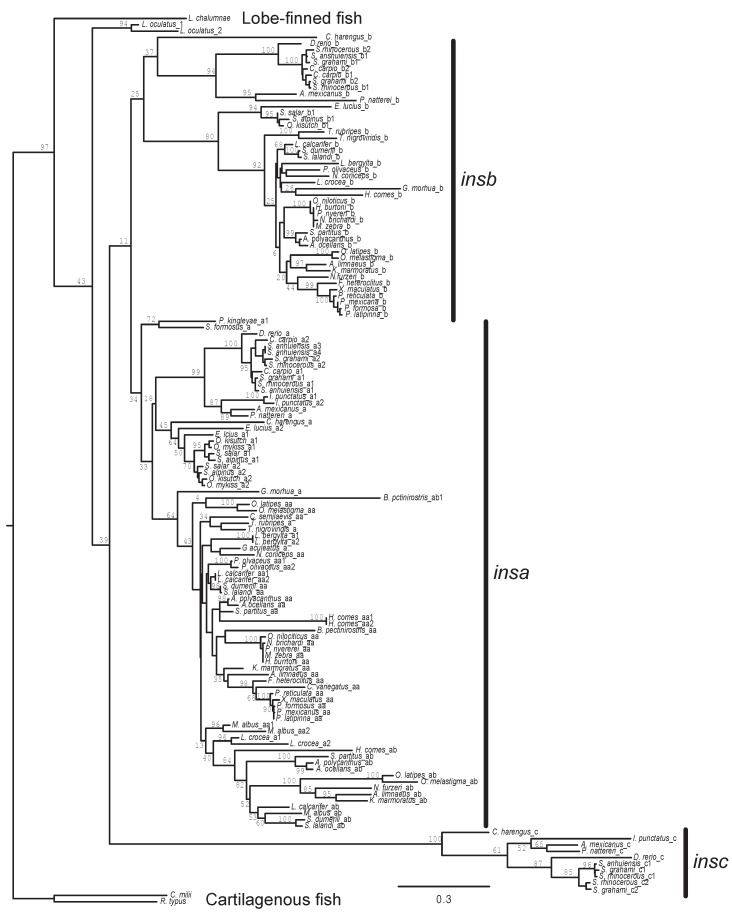
Phylogeny of fish insulin ge

To better understand the origin and evolution of the multiple insulin (*ins*) genes found in the genomes of ray-finned fish, their phylogenetic relationships, rooted using *ins* gene sequences from cartilaginous and lobe-finned fish, were inferred using maximum likelihood (Figure 2) and Bayesian (Supplementary Figure S2) methods. Both analyses generated similar phylogenies with independent duplications of the insulin gene on the spotted gar (*Lepisosteus oculatus*; infraorder Holostei) and teleost (infraorder Teleostei) fish lineages. While duplicated *insa* and *insb* genes were previously identified in teleost fish (Caruso & Sheridan, 2014; Hrytsenko et al., 2016; Irwin, 2004), here I find that these species have a third insulin paralog, which I named *insc*. The species relationships within each paralog of insulin genes, given its limited power at resolving species relationships, is in general agreement with the accepted phylogeny of teleost fish (Betancur-R et al., 2017; Near et al., 2012). This observation suggests that the triplication of the insulin gene occurred in an early teleost. The relationships among the *insa*, *insb*, and *insc* paralogs is poorly resolved in our phylogenetic analyses, with the maximum likelihood analysis suggesting a closer relationship between *insa* and *insb*, while the Bayesian analysis suggesting that *insb* and *insc* are most closely related.

Phylogenetic relationship of insulin coding sequences was inferred using maximum likelihood with the best fitting evolutional model GTR+G+I (GTR with gamma distribution and invariant sites) and bootstrapped with 500 replications. A similar phylogeny was generated using Bayesian methods (Supplementary Figure S2). Percent bootstrap support for lineages are shown. The tree in Newick format, with all branch lengths and support values, is presented in Supplementary Figure S3. The phylogeny was rooted with sequences from cartilaginous (*Callorhinisus milli* and *Rhincodon typus*) and lobe-finned (*Latimeria chalumnae*) fish. Full species names are listed in Figure 1. *insa*, *insb*, and *insc* indicate the three clades of insulin genes found in teleost fish.

Most teleost species that possess *insb* and *insc* genes have a single copy of each gene, except those species that have experienced additional genome duplications (i.e., *Cyprinus carpio*, salmonids (*O. mykiss*, *O. kisutch*, *Salmo salar*, and *Salvelinus aplinus*), and the genera *Sinocyclocheilus*)). In contrast, a large number (30 of 51) of teleosts have multiple *insa* genes (Figure 1 and Supplementary Table S1). While some of these appear to be due to lineage-specific genome duplications (i.e., *Cyprinus carpio*, salmonids, and the genera *Sinocyclocheilus*) or single-gene duplications (e.g., *Labrus bergylta* and *Larimichthys crocea*), many are due to a duplication event that occurred on an early Percomorpha lineage (Figure 1, Figure 2, and Supplementary Figure S2). The genomes of many Percomorpha fish contain two (or more) *insa* genes that form monophyletic clades that are largely in accord with the accepted species phylogeny (Betancur-R et al., 2017; Near et al., 2012). This observation suggests that a duplication of the *insa* gene, generating *insaa* and *insab* genes, occurred in an early lineage of Percomorpha fish (see Figure 1). In species that are inferred to be descendants of this Percomorpha-specific *ins* gene duplication, all but *Nothobranchius furzeri* possess an *insaa* gene, however, a larger number of species, such as tilapia (*Oreochromis niloticus*), some cichlids (e.g., *Pundamilia nyererei* and *Maylandia zebra*), and species of the genus *Poecilia* do not have an *insab* gene (Figure 1, Figure 2 and Supplementary Figure S2 and Table S1). This suggests that the *insab* gene was lost in parallel in a number of species of Percomorpha (see Figure 1).

### Duplications of fish insulin genes

**Figure 3:**
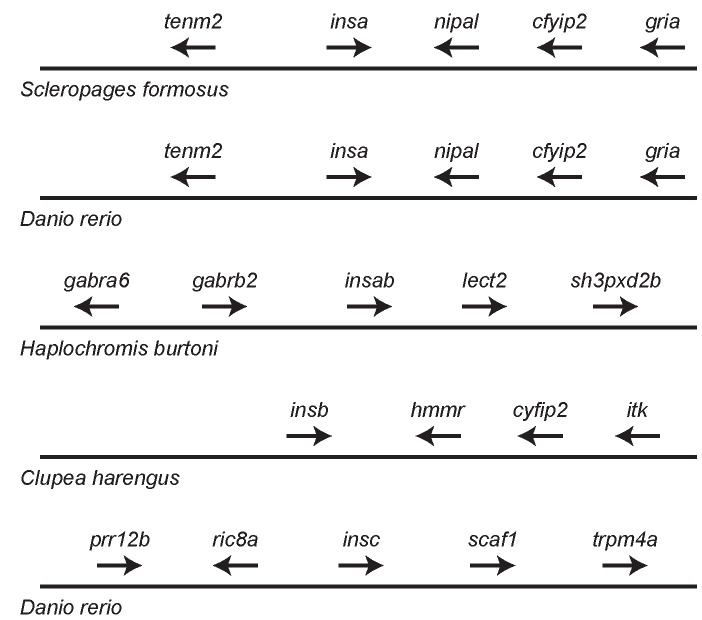
Genomic neighborhoods surrounding fish insulin gene

To aid in the assessment of the orthology of insulin genes, and to gain insight into the gene duplication events, I identified genes flanking the insulin genes in the genomic sequences of 55 species of fish (Supplementary Table S2, examples given in Figure 3). A genome duplication occurred in the ancestor of teleost fish, the teleost fish-specific genome duplication (Glasauer & Neuhauss, 2014; Meyer & Van de Peer, 2005). Our phylogenetic analyses (Figure 2 and Supplementary S2) suggests that the three insulin paralogs found in teleost fish (*insa*, *insb*, and *insc*) originated on this lineage. If duplicate genes originated through a genome duplication, then one might expect similar genomic neighborhoods for these genes (i.e., similar genes found adjacent to different *ins* genes), as observed for the duplicated proglucagon (*gcg*) and glucagon receptor (*gcgr*) genes of teleost fish (Irwin, 2014; Irwin & Mojsov, 2018). For the three insulin paralogs examined here, only very limited similarity was seen in the genomic neighborhoods (longer genomic regions were examined, with immediate neighborhoods summarized in Supplementary Table S2). A gene similar to *tenm2* (teneurin transmembrane protein 2) was found adjacent to *insa* genes in *Exox lucius*, *O. kisutch*, *O. mykiss*, *Paramormyrops kingsleyae*, *Salmo salar*, and *Sclerophages formosus*, and *insb* genes in *Pygocentrus nattereri*, *Salvelinus alpinus*, and *Sinocyclocheilus rhinocerous* (Supplementary Table S2). This might suggest that the *insa* and *insb* genes originated via the teleost-fish specific genome duplication. However, as the linkage between the *tenm2* and *ins* genes was only observed in a small number of species, this would require large number of independent losses (or genomic rearrangements) of *tenm2*-like genes to yield the absence of a linked *tenm2*-like with most *ins* genes, therefore the evidence for the origin of *insa* and *insb* by a genome duplication event is not strong. No genes near *insc* were similar to those adjacent to *insa* or *insb*, thus it more likely originated as a single gene duplication event rather than through a genome duplication. If the *insa* and *insb* genes originated via a genome duplication, an addition single-gene duplication would still be needed to explain the origin of *insc*. The gene neighborhood data does not help resolve the relationships among the three *ins* genes. Intriguingly, the genomic neighborhoods of none of the insulin gene paralogs in teleost fish is similar to the typical vertebrate insulin gene neighborhood, where insulin is flanked by insulin-like growth factor 2 (*igf2*) and tyrosine hydroxylase (*th*) genes (Patton et al., 1998), although similar neighborhoods were found for the insulin genes in cartilaginous and lobe-finned fish (Supplementary Table S2). This suggests that the insulin genomic neighborhood was rearranged on the ancestral ray-finned fish lineage.

Examples of genomic neighborhoods found for insulin paralogs in ray-finned fish. Genomic neighborhoods are listed in Supplementary Table S2. A: Typical genomic neighborhood for *insa* and *insaa* genes as illustrated from *Sceleropages formosus*. B: Genomic neighborhood for *insa* genes in *Danio rerio* and close relatives. C: Typical genomic neighborhood for *insab* genes as illustrated from *Haplochromis burtoni*. D: Typical genomic neighborhood for *insb* genes as illustrated from *Clupea harengus*. E: Typical genomic neighborhood for *insc* genes as illustrated from *D. rerio*. See Supplementary Table S2 for full gene names.

Orthologous genes often have similar genomic neighborhoods, thus can these neighborhoods can be used to assess orthology when gene sequence similarity is limited (Kurokawa et al., 2005). When the genomic neighborhoods surrounding *insb* and *insc* genes were examined (Figure 3 and Supplementary Table S2) shared features were identified for each gene. All *insb* genes are adjacent to a *hmmr* (hyaluronan mediated motility receptor)-like gene, while all *insc* genes are flanked by *prr12* (proline rich 12) and *scaf1* (SR-related CTD associated factor 1)-like genes (as shown for the *Clupea harengus insb* and *Danio rerio insc* genes in Figure 3), suggesting that their genomic neighborhoods are largely conserved since they originated in the early teleost. In contrast, several different genomic neighborhoods were observed for the *insa* genes (Supplementary Table S2, examples shown in Figure 3). The most phylogenetically widespread genomic neighborhood has a *nipal* (magnesium transporter NIPA3-like)-like gene adjacent to the *insa* gene (Supplementary Table S2) and is found in species representing Osteoglossomorpha (e.g., *Scelerophages formosus*, shown in Figure 3), Protacanthopterygii (e.g. *Esox lucius* and *Salmo salar*), Paracanthopterygii (e.g., *Gadus morhua*), and Percomorpha (e.g., *Takifugu rubripes* and *Oryzias latipes*). The only major group of teleost fish that does not have an *insa* gene adjacent to *nipal* are those of Otomorpha (e.g., *Danio rerio*, shown in Figure 3), suggesting a change in the gene order occurred in this group and that the linkage of *insa* with *nipal* is ancestral. Within Percomorpha (Supplementary Table S2), the *insaa* genes are found adjacent to *nipal*-like genes (as in *Scelerophages formosus*, Figure 3), while the *insab* genes are near a *sh3pxd2b* (SH3 and PX domains 2B)-like genes (e.g., *Haplochromis burtoni*, Figure 3). The linkage of the *insab* genes with *sh3pxd2b* strengthens the conclusion that all *insab* genes are orthologous and indicates that *insaa* is located at the ancestral genomic location and was the source (locus-of-origin) for the *insab* gene that was inserted into a new genomic locus.

### Lineage-specific changes in insulin gene number

The gene duplication events described above explain most, but not all, of the insulin genes identified (Figure 1 and Supplementary Table S1). Many of the additional genes can be explained by tandem gene duplication, which lead to two insulin genes arranged head-to-tail in the genome (see Supplementary Tables S1, S2) and includes the *insa1* and *insa2* genes in *Parmormyrops kingsleyae* and in *Larmichthys crocea*, the *insaa1* and *insaa2* genes in *Hippocampus comes*, and the *insab1* and *insab2* genes in *Boleopththalmus pectinirostris*. Both *Cyprinus carpio* and *Sinocyclocheilus rhinocerous* have tandemly arranged *insb* genes (*insb3* and *insb4* in *C. carpio* and *insb2* and *insb3* in *S. rhinocerous*, Supplementary Table S2) that might have originated prior to the divergence of these two species (although, they would then have to be lost in the two other *Sinocyclocheilus* species). *Sinocyclocheilus anshuiensis* has two pairs of tandemly arranged genes, *insa1*/*insa2* and *insa3*/*insa4*, which either originated prior to this species genome duplication (and then were lost in the closely related species that share this genome duplication), had parallel tandem duplications, or is an assembly error (the tandemly arranged genes are identical in sequence). The *ins1* and *ins2* genes of *Lepisosteus oculatus* might also have originated via a tandem duplication, which was then followed by rearrangements so that these lineage-specific gene duplicates are now found in opposite orientations on either side of a *hmmr*-like gene (see Supplementary Table S2). More complex duplication events, resulting in genes moving to new locations are need to explain the remaining genes, with some being extremely recent (e.g., the identical *insaa1* and *insaa2* genes in *Labrus bergylta*) or more ancient (e.g., *insa1* and *insa2* gene of *Esox lucius*).

While duplication of genomic DNA is a frequent mechanism for the origin of duplicate genes, they can also be generated by retroposition of cDNA generated from mRNA transcripts (Long et al., 2013). Indeed, the well-characterized duplicate *ins1* gene in mice was generated from an incompletely processed mRNA transcript (Shiao et al., 2008; Soares et al., 1985). Typically, insulin genes contain two coding exons that must be spliced together to create an intact coding sequence. Here I identified 6 insulin genes that are likely generated by reverse-transcribed mRNAs, as these genomic sequences do not have an intron interrupting the coding sequence (see Supplementary Figure S4 for an example). The 6 retro-processed insulin genes can be explained by three integration events: (1) between *ankrd6* (ankyrin repeat domain 6) and *lyrm2* (LYR motif containing 2)-like genes on the *Lepisosteus oculatus* lineage to generate ψ*ins*; (2) within an *apbb3* (amyloid beta precursor protein binding family 8 member 3)-like gene on the *Monopterus albus* lineage to generate ψ*insb*; and (3) within a *psd3* (PH and SEC7 domain containing protein 3)-like gene on the ancestral lineage for salmonids (*Oncorhynchus mykiss*, *O. kisutch*, *Salmo salar*, and *Salvelinus aplinus*) and the Northern pike (*Esox lucius*) that generated ψ*insa* in *O. kisutch*, *S. salar*, and *S. alpinus* (but was not found in *O. mykiss*) and an intact coding sequence (*insa2*) in *Esox lucius* (Figure 1 and Supplementary Figure S4 and Table S2). The maintenance of an intact open reading frame in *Exox lucius insa2* (Supplementary Figure S4) suggests that this retropositioned sequence, like the mouse *ins1* gene (Soares et al., 1985), is still functional. However, this sequence has lost its coding potential in salmonid fish and is a pseudogene (Figure 1 and Supplementary Table S1). Additional processed genes might also exist, as several incomplete genes (e.g., *insb4* from *Cyprinus carpio* and *insa3* from *Paramormyrops kingsleyae*) were identified (see Supplementary Table S1) that were located at unique location of the genome (see Supplementary Table S2) consistent with being inserted processed cDNAs, but the sequences were similar to either exon 1 or 2 (and not both), thus cannot be distinguished from a sequence generated by an DNA-mediated incomplete gene duplication event.

### Gene for insulin a (*insa*) is evolving under greater evolutionary constraint

Our genomic and phylogenetic analyses of fish genome sequences demonstrated that teleost fish have three paralogous insulin genes. In contrast, most other vertebrate species only have one (Chan & Steiner, 2000; Conlon, 2000b). The increased number of insulin genes in teleost fish raises the possibility that they have been diversified to: (1) subfunctionalize distinct functions of insulin, (2) neofunctionalize to acquire novel functions, or (3) lose function (pseudogenize). As a first step to explore possible changes in the biological roles of these distinct insulin genes I assessed the selective pressures acting upon the sequences. This can be done by comparing the rates of nonsynonymous (*d*_N_) to synonymous (*d*_S_) substitutions (Yang & Bielawski, 2000). If the protein encoded by a gene has lost its function, then it would be expected that there would be no selection against nonsynonymous substitutions, and that rates of nonsynonymous and synonymous substitutions would be the same rate (i.e., *d*_N_/*d*_S_=1, neutral evolution). If the protein encoded by these genes still had a function, then selection should act against a subset of nonsynonymous substitutions (the deleterious mutations) and be lower than the synonymous rate (i.e., *d*_N_/*d*_S_<1, purifying selection). Rarely, one might see *d*_N_/*d*_S_>1, which would indicate positive selection for change in amino acid sequence (Yang & Bielawski, 2000). If protein coding sequences of genes are being maintained for different functions, with different parts of the sequence being important for these functions, then one might see differing levels of selective constraint.

I used the program RELAX (Wertheim et al., 2014) to assess the levels of selective pressure (*d*_N_/*d*_S_) for each of the three insulin gene paralogs, and to determine whether the selective pressure was intensified or relaxed compared to the other insulin genes, with sequences from a non-teleost ray-finned fish, *Lepisosteus oculatus*, used as outgroup. When all sequences were analyzed, *insa* genes showed the stronger levels of purifying selection (*d*_N_/*d*_S_=0.266 6, 0.333 2, and 0.325 8 for *insa*, *insb*, and *insc*, respectively; Table 1). If I restricted these analyses to either species that have all three paralogs, or species that only have a single copy of these paralogs, to attempt to minimize species-specific effects (changes seen in species that only have some of the genes) or due to differences in gene number, the difference in the selective constraints acting on *insa* vs. *insb* and *insc* became even more pronounced (Table 1). The results from RELAX also indicated that selection intensification, compared to the other insulin sequences, occurred on *insa*, while selection relaxation occurred for *insb* (Table 1). No significant change in selection intensity was seen for *insc* vs. all other insulin sequences. Similar results, with *insa* showing greater constraint, were seen in comparisons of *insa* and *insb* genes in species that had both of these genes (Table 1). These results suggest that the *insa* paralog is under greater purifying selective constraint than either the *insb* or *insc* paralog, but also importantly demonstrate that both *insb* and *insc* are continuing to experience purifying selection. Thus, it can be concluded that the protein coding sequence of *insa* is under the greatest level of selective constraint, however, the coding sequences of both *insb* and *insc* are also experiencing selective constraints consistent with their protein products having essential biological functions.

**Table 1:** Differences in the selective pressure (*d*_N_/*d*_S_) acting on insulin paralogs in teleosts

Sequences tested^a^	*insa*	*insb*	*insc*
All	0.2666^b^ (80)	0.3332^c^ (44)	0.3258 (10)
Species with *insa*, *insb*, and *insc*	0.2120^b^ (11)	0.3309^c^ (9)	0.3406 (9)
Species with single copy *insa*, *insb*, and *insc*	0.1911 (4)	0.3164 (4)	0.3129 (4)
Species with single copy *insa* and *insb*	0.1664^b^ (17)	0.3171^c^ (17)	N/A
Species with single copy *insa* and *insb*, but no *insc*	0.1366^b^ (13)	0.3191^c^ (13)	N/A

Numbers in brackets is the numbers of sequences used. ^a^: The two complete *Lepisosteus acaulatus ins* sequences were used as outgroups. ^b^: Test for selection intensification was significant. ^c^: Test for selection relaxation was significant. N/A: Not applicable.

### Adaptive evolution of insulin genes

While the protein products of the three insulin genes are being maintained for function, they might not be the same function. To examine whether any of the sequences might have gained new functions I tested for evidence of positive selection on lineages using aBSREL (Smith et al., 2015). Only one lineage showed significant evidence for episodic diversifying selection, the ancestral lineage for the *insc* genes. Inspection of the phylogenetic tree generated from all of the insulin coding sequences suggest that the ancestral branch for the *insc* genes is longer than for the *insa* and *insb* genes (Figure 2 and Supplementary Figure S2), consistent with a greater number of nonsynonymous substitutions driven by positive selection. No evidence for positive selection was found on the ancestral lineages for the *insa* or *insb* genes. These results might suggest that *insc* has acquired a new function (neofunctionalization) driven by positive selection, while *insa* and *insb* have been retained due to subfunctionalization of insulin functions between the two genes.

### Relaxed selection on the *insab* duplicate of the insulin a (*insa*) gene

An intriguing observation from our analysis of the selective constrains acting upon *insa*, *insb*, and *insc* paralogs was that the calculated value for the selective constraint acting on *insa* varied considerably depending upon which sequences were used for the analysis (Table 1). The ratio of the nonsynonymous to synonymous rates was higher when all *insa* sequences were used than if sequences were used only from species that had all three paralogs or had only single copies of the *insa* and *insb* paralogs (Table 1). These observations suggest that inclusion of *insa* sequences from species that have multiple *insa* sequences yields higher estimates of the *d*_N_/*d*_S_ ratio due to some of these sequences having lower levels of sequence constraint. As the duplication to generate the *insaa* and *insab* genes is a major source of the multiple *insa* genes in teleost fish (see Figure 1, Figure 2 and Supplementary Figure S2 and Table S1), I compared the selective constraints acting upon *insaa* and *insab* genes (Table 2). The *insaa* coding sequences were found to be under selective constrains (Table 2) similar to those of other *insa* sequences, and especially those from species that had single copy *insa*, while the *insab* sequences displayed the lowest levels of constrains seen for any fish *ins* sequence (Table 1, Table 2). Test for intensification or relaxation of selective constraint showed that *insaa* was under significant intensification of selective constraint, while *insab* was significant relaxation of selective constraint was demonstrated for *insab* (Table 2). These results suggest that *insaa* retains the function of *insa*, which would be consistent with it being at the locus of origin, while *insab* is evolving with far less constraint, to the extent that it has been lost on a number of lineages (e.g., *Oreochromis niloticus*, *Pundamilia nyererei*, *M. zebra*, and species of the genus *Poecilia*, see Figure 1). This might suggest that *insab* is not essential.

**Table 2:** Differences in the selective pressure (*d*_N_/*d*_S_) acting on insaa and insab genes

Sequences tested^a^	*insaa*	*insbb*
All *insa*	0.1691^b^ (18)	0.4766^c^ (14)
Species with one copy of each	0.1251^b^ (9)	0.5139^c^ (9)

Numbers in brackets is the numbers of sequences used. ^a^: The two complete *Lepisosteus acaulatus ins* sequences were used as outgroups. ^b^: Test for selection intensification was significant. ^c^: Test for selection relaxation was significant.

### Processing of proinsulin sequences

To be functional, the protein product encoded by insulin genes need to be proteolytically processed and secreted to generate two-peptide chain insulin molecules (Steiner et al., 1996, 2009). Our analysis was initially only focused on sequences that showed similarity to previously characterized proinsulin sequences and had intact coding sequences, i.e., had an initiation codon, a termination codon and intact open reading frame with greater similarity to proinsulin than to other insulin-like sequences (e.g., insulin-like growth factors). To determine whether the encoded protein sequences could be secreted and properly processed I searched for potential signal peptidases and proprotein processing sites using programs that predict these sites (Duckert et al., 2004; Petersen et al., 2011; Southey et al., 2006).

The coding sequences of intact proinsulin open reading frames from all ray-finned fish identified here are predicted to have functional signal peptides (Table 3 and Supplementary Table S3 and Figure S6), thus should be able to be secreted. In addition, all of the proinsulin protein sequences encoded by the *insa*, *insab*, *insb*, and *insc* genes have potential prohormone protease cleavage sites that could yield typical two-chain insulin hormone molecules (Table 3 and Supplementary Table S3 and Figure S6). In contrast, however, only one of the 14 proteins encoded by *insab* genes is predicted to potentially produce a two-chain insulin (Table 3). For the insab sequences, only 2 have potential B-chain/C-peptide processing site and 9 have potential C-peptide/A-chain processing sites (Table 3 and Supplementary Table S3 and Figure S6), indicating that neither site is conserved. Both NeuroPred (Southey et al., 2006) and ProP (Duckert et al., 2004) predicted similar sites, but ProP also provided a list of other potential processing sites that did not score well enough to be predicted sites (see Supplementary Table S3). The alternative potential sites identified by ProP in the insab proteins still would not generate 2-chain insulin molecules similar to functionally characterized insulin molecules (Steiner et al., 1996, 2009) as they would generate very short B-chains, often missing residues essential for function.

**Table 3:** Secretion and processing of fish proinsulin proteins

Gene	Signal	B-C	C-A	2-chain
	peptide^a^	junction^b^	junction^b^	insulin^c^
*insa*	35/35^d^	35/35	35/35	35/35
*insaa*	32/32	32/32	32/32	32/32
*insab*	14/14	2/14	9/14	1/14
*insb*	45/45	44/45	45/45	44/45
*insc*	10/10	10/10	10/10	10/10

^a^: Sequences with signal peptidase processing sites. ^b^: Sequences with proprotein processing sites for the B-chain/C-peptide and C-peptide/A-chain processing sites. ^c^: Number that can be secreted and properly processed to yield two-chain polypeptides. ^d^: Number with the site/total number of sequences.

**Figure 4:**
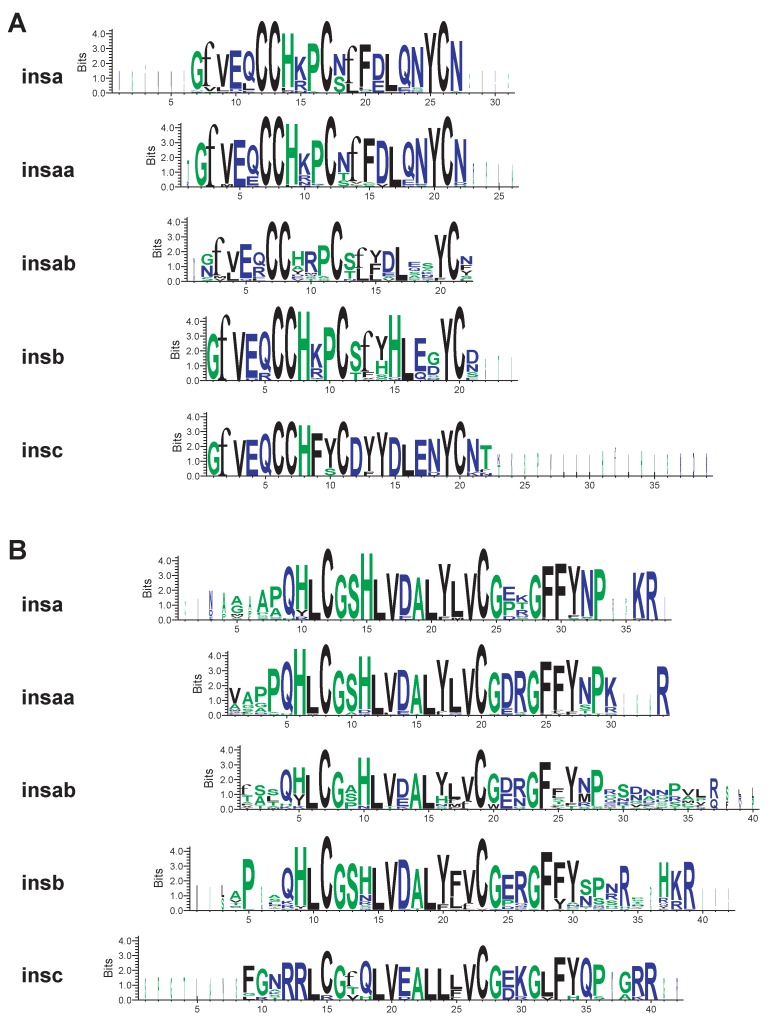
Consensus fish insulin peptide sequences

The hormone insulin is a two-chain peptide molecule held together by disulfide bridges (Steiner et al., 1996, 2009). To confirm that two-chain molecules could be produced from the proinsulin protein sequences I generated consensus sequences for the A- and B-chains for the different types of insulins (Figure 4). The lengths of the predicted A- and B-chains were similar between the different types of insulins, with more variation, at both the N- and C-termini, within proteins encoded by a type of gene than between types of genes (Figure 4 and Supplementary Figure S6). When cysteine residues were examined one of the two insc sequences from *Sinocyclocheilus grahami* (*insc2*) had a cystine replaced by an arginine residue. It is possible that the *S. graham insc2* gene does not encode a functional insulin, but this is compensated by the presence of an *insc1* gene that encodes an insulin that can be secreted processed and has all six cysteine residues. Surprisingly, when I examined A- and B-chain sequences homologous to those of other insulins in the protein encoded by *insab* genes, all of the cysteine residues were perfectly conserved, however much of the rest of the sequence showed greater variation than within other types of insulins (Figure 4 and Supplementary Figure S6). This raises the possibility that insab could still fold in a similar fashion as typical insulin, thus be transported to insulin secretory granules, but then not be processed into an active hormone (Liu et al., 2015).

Consensus sequences for the A-chain (A) and B-chain peptides (B) for insulin encoded by *insa*, *insaa*, *insab*, *insb*, and *insc* genes. Consensus sequences were generated from aligned predicted protein sequences (see Supplementary Figure S5) of genes with complete open reading frames (*Larimichthys crocea insa2* was removed as it may not be functional). Hight of the residue is proportional to representation among the compared sequences, while residue width represents proportion of sequences without a gap (thin residues are gaps in many sequences).

## DISCUSSION

Searches of diverse fish genomes, including in the extensively characterized zebrafish (*Danio rerio*) genome, unexpectedly revealed the existence of three insulin gene paralogs (Figure 1 and Supplementary Figure S1 and Table S1). Previously, two paralogous insulin genes, *insa* and *insb*, had been found in the zebrafish genome that have distinct expression patterns (Irwin, 2004; Papasani et al., 2006). Here I found *insa* genes in the genomes of all teleost fish (Figure 1 and Supplementary Figure S1 and Table S1), which encode the previously isolated insulin hormone sequences (Caruso & Sheridan, 2011; Conlon, 2000b) that regulate glucose metabolism. Given the central role of insulin in the regulation of metabolism (Caruso & Sheridan, 2011; Röder et al., 2016; Weiss, 2009), it is not surprising that this gene was found in all fish. The presence of a high selective constraint (Table 1, Table 2) on the protein sequences encoded by *insa* and *insaa* genes supports the conclusion that this sequence is essential. A surprising discovery was the presence of a duplicate of the *insa* gene, the *insaa* and *insab* genes, in a large number of teleost fish (Figure 1 and Supplementary Table S1). The protein coding sequence encoded by *insab* genes have the highest nonsynonymous substitution rate (Table 2), and thus least selective constraint, yet the consensus sequences of the potential A- and B-chains of insulin predicted from these genes have perfectly conserved cystine residues (Figure 4). This suggests that these genes might encode proinsulin-like proteins that could properly fold but would not be properly processed in secretory granules to produce active insulin molecules (Liu et al., 2015). Thus, insab proteins might compete with insaa proteins for processing enzymes and modulate the release of functional insulin molecules in these species.

The *insb* gene was found in the genomes of almost all teleost fish species examined, demonstrating that it is conserved in these fish (Figure 1 and Supplementary Figure S1 and Table S1). The selective forces acting on *insb* are weaker than those acting on *insa* (Table 1) suggesting that these two genes have different functions. Previous work indicates that *insb* is not appreciably expressed in the pancreas, and instead is more predominantly expressed in early development (Hrytsenko et al., 2016; Irwin, 2004; Papasani et al., 2006). The analysis of selective constraints and these expression results are consistent with speculation that *insb* is not primarily involved in the regulation of blood glucose level, but instead has a role in development (Hrytsenko et al., 2016; Papasani et al., 2006). Given the conservation of the *insb* gene across teleost fish, it likely acquired this role soon after its origin and has been conserved.

Unexpectedly I found an *insc* paralog, and it was present in the well characterized zebrafish (*D. rerio*) genome (Figure 1 and Supplementary Figure S1 and Table S1). Upon closer examination, the *insc* gene in both the Ensembl (gene ID: ENSDARG00000096862) and NCBI (GenBank accession No.: 100534937) zebrafish genomes fail to predict an intact open reading frame, with the intact *insc* coding sequence only coming from the linked mRNA sequence (XM_009299616.3) in the NCBI database (see Supplementary Table S1). I could find no raw sequence data (e.g., ESTs) that supports the existence of an intact open reading frame for zebrafish *insc*, as all mRNA derived sequences (GenBank accession Nos. EH533807.1 and EH507658.1, forward and reverse sequences of the same EST clone) also contained the deletion found in the genomic sequence. This suggests that the zebrafish *insc* gene does not encode an intact protein product. However, our analysis of the selective forces acting upon the *insc* coding sequences indicate that the nonsynonymous rate of substitutions (*d*_N_) is much lower than the rate of synonymous substitutions (*d*_S_) consistent with selection to maintain a protein coding sequence (Table 1) in most, if not all, species that have intact *insc* genes. The *insc* gene, in contrast to *insa* and *insb*, is restricted to the genomes of only a few lineages, with this gene been lost from the genome of most teleost fish (Figure 1 and Supplementary Table S1). This distribution of the gene among fish suggests that it does not encode a function that is essential in most species, and its loss might have been compensated by the presence of paralogs of insulin in the fish genomes. Expression of *insc*, based on a single EST clone (with sequences from both ends) that was identified in zebrafish, is found in the “gut and internal organs” of adults, thus potentially it could be expressed in the pancreas and be replaced by *insa*. Further studies on the expression of *insc* in other fish are needed to identify its role in physiology.

## CONCLUSION

Searches of ray-finned fish genomes have demonstrated that they contain more insulin genes than previously appreciated and suggest that the roles of these genes have diversified. The diversification of the functions of insulin parallels the diversification of the proglucagon-encoded peptides found in fish (Irwin & Mojsov, 2018). Similarly, parallel changes in the biological functions of insulin and proglucagon-derived peptides have been observed in hystricomorph rodents (i.e., guinea pig and relatives), where changes in sequence and action of insulin have compensating changes in glucagon (Seino et al., 1988).
